# Subclinical Scores in Self-Report Based Screening Tools for Attention Deficits Correlate With Cognitive Traits in Typical Evening-Type Adults Tested in the Morning

**DOI:** 10.3389/fpsyg.2019.01397

**Published:** 2019-06-18

**Authors:** Maria Korman, Ishay Levy, Rinatia Maaravi-Hesseg, Adi Eshed-Mantel, Avi Karni

**Affiliations:** ^1^The Edmond J. Safra Brain Research Center for the Study of Learning Disabilities, University of Haifa, Haifa, Israel; ^2^Laboratory for Human Brain and Learning, Sagol Department of Neurobiology, University of Haifa, Haifa, Israel; ^3^Department of Occupational Therapy, Faculty of Social Welfare and Health Sciences, University of Haifa, Haifa, Israel; ^4^FMRI Unit, Diagnostic Radiology, The Chaim Sheba Medical Center, Tel Hashomer, Israel

**Keywords:** chronotype, attention deficits, day-time sleepiness, alertness, reaction time, executive functioning, actigraphy

## Abstract

Previous studies suggest that in adolescents and young adults, evening chronotype is a subclinical factor in physical, cognitive, and psychiatric fitness; poor sleep habits and larger misalignment with the social schedule constraints may exacerbate symptoms of inattention, impulsivity and the risks for detrimental behaviors. The influence of chronotype on neurocognitive performance during morning hours and scores in self-reports about attention deficit symptoms (ADS) and executive functioning, was explored in 42 healthy young university students (29 women), divided to evening type (ET) and combined morning/intermediate type (MT/IT) groups. Evening chronotypes scored significantly higher in the questionnaires of inattention Adult ADHD Self-Report Scale (ASRS-6) (MT/IT: 1.62 ± 1.59; ET: 2.71 ± 1.62, *p* < 0.05) and day-time sleepiness Epworth scale (MT/IT: 7.19 ± 5.17; ET: 11.48 ± 5.26, *p* < 0.01), reported lower subjective alertness (MT/IT: 63.02 ± 21.40; ET: 40.76 ± 17.43, *p* < 0.001), and had slower reaction times (MT/IT: 321.47 ± 76.81; ET: 358.94 ± 75.16, *p* < 0.05) during tests, compared to non-evening chronotypes. Nevertheless, ETs did not significantly differ in self-reports of executive functioning in the Behavioral Rating Inventory of Executive Functions-A (BRIEF-A) from non-ETs. The scores on standard self-report screening tools for ADS and executive functioning (ASRS-6, BRIEF-A-Metacognition) correlated with eveningness. We conclude that eveningness, subjective sleepiness and low arousal levels during morning can present as subclinical Attention Deficit and Hyperactivity Disorder (ADHD) symptoms in typical young adults with no evident sleep problems. Self-report based screening tools for ADS and executive functioning reflect chronotype-related traits in healthy young adults. Strong eveningness may bias the results of neurocognitive performance screening for ADHD when administered at morning hours.

## Introduction

Circadian rhythms exhibit a high inter-individual variability and are classified to three main types along a continuum from morningness to eveningness, based on the preferred timing of sleep-wake activity (Horne and Ostberg, 1976; Adan et al., 2012): MT, IT, and ET. Circadian profiles of melatonin and cortisol secretion (Bailey and Heitkemper, 2001; Lack et al., 2009), core body temperature (Vitiello et al., 1986) but also alertness (Matchock and Mordkoff, 2009; Barclay and Myachykov, 2017) and meal timing (Rossbach et al., 2018), were found to be associated with each chronotype. Genetic variations in circadian genes prompt the propensity of each individual’s circadian rhythm to be entrained by light (Harvey, 2008), however, the timing of sleep is dependent on additional factors, including accumulated sleep debt and light exposure and social schedules (Roenneberg et al., 2003). In adults, an individual’s chronotype may be reliably estimated using self-report inventories, such as the Horne–Ostberg MEQ (Horne and Ostberg, 1976; Roenneberg et al., 2003).

Chronotypes differ beyond variations in circadian timing, including differences in personality and cognitive traits (Adan et al., 2012), as well as health-related disparities (Caci et al., 2004; Adan et al., 2010, 2012; Roenneberg and Merrow, 2016). For example, individuals with MT may tend to have higher conscientiousness and agreeableness (Adan et al., 2012) and may perform better on cognitive and mood regulation tasks compared to ET (Harvey, 2008; Kalmbach et al., 2017). Different chronotypes exhibit significant relative shifts in times of highest/lowest alertness and performance abilities (Bennett et al., 2008; Hahn et al., 2012). MTs tend to reach their best performance levels in the morning, while ITs and ETs in the afternoon, a “synchrony effect” (Adan et al., 2012). This effect was suggested to be explained by the co-occurrence of peaks in alertness, core body temperature, and arousal.

Circadian fluctuations in arousal may have a critical impact in everyday situations that require emotion-independent, sustained, effortful cognition. Executive functions (EFs) conceptualized as a set of higher-order cognitive abilities involved in the organization, direction, and management of cognitive resources, as well as of emotional responses, are particularly sensitive to the time-of-day (Miyake et al., 2000; Schmidt et al., 2007; Diamond, 2013). Specifically, inhibitory control is lower during reduced subjective alertness (May, 1999), while peaks in inhibitory control correspond to peaks in subjective alertness, variations that relate to chronotype (Manly et al., 2002).

People with evening chronotypes are prone to misalignments between socially defined sleep-wake schedules and physiologically driven preference for later timing of sleep, leading to chronic shortening of sleep duration on work days (McGowan et al., 2016; Roenneberg and Merrow, 2016). Sleep deprivation and sleep restriction are ubiquitous among college students (Hicks et al., 2001; Lund et al., 2010), typically belonging to the age group with the highest frequency of evening chronotypes (Roenneberg et al., 2003). Sleep-loss is related to cognitive deficits in healthy adults, including altered emotionality (Horne and Norbury, 2018), attention and executive control (Anderson et al., 2009; Rossa et al., 2014; Cunningham et al., 2018), and ADHD – like traits, such as unstable and disinhibited temperaments (Ottoni et al., 2012; Park et al., 2015) and sensation-seeking behavior (Hsu et al., 2012; Kang et al., 2015). Delayed sleep phase syndrome, eveningness, and sleep disorders are often comorbid in adults and adolescents with ADHD (Gruber et al., 2007; Moreau et al., 2013; Owens et al., 2013; Wajszilber et al., 2018). Shortened, mistimed, sleep was suggested to mediate the impact of evening chronotype on ADHD traits in the healthy young adult population (Gruber et al., 2012; McGowan et al., 2016; McGowan and Coogan, 2018).

Deficits in the EFs appear to be an important component of the complex neuropsychology of ADHD (Doyle, 2006; Marzinzik et al., 2012; Korman et al., 2018; Silverstein et al., 2018), contributing to problems in everyday functioning and psychological well-being. ADHD in adolescents and adults is often associated with the evening chronotype (Kooij and Bijlenga, 2013; Bumb et al., 2016; Coogan and McGowan, 2017; Vogel et al., 2017), dysregulation of arousal during wake (Brennan and Arnsten, 2008; Hegerl and Hensch, 2014) and sleep disturbances (Um et al., 2017; Korman et al., 2018). Low arousal during wake was suggested to underlie deficits in attention in ADHD (Sikstrom and Soderlund, 2007; Caci et al., 2009; Strauss et al., 2018).

The diagnosis of ADHD relies heavily on subjective assessments of perceived behavior (Gualtieri and Johnson, 2005). For adults, several major self-rated scales are widely used to assess whether the criteria necessary for a diagnosis of ADHD according to the Diagnostic and Statistical Manual of Mental Disorders (DSM-5 314.00, 314.01), are met (Goodman, 2009); these include the World Health Organization ASRS-6 (Kessler et al., 2005) and the BRIEF-A (Roth et al., 2005). ASRS-6 score was recently found to be correlated with circadian typology (McGowan et al., 2016).

These considerations suggest that an investigation of the interactions between individual differences in circadian variability (chronotype and arousal) and the subjective and objective level of executive functioning using screening tools designed to detect ADHD symptoms is appropriate for healthy adults without ADHD. Thus, the aim of the present study was to assess differences between young healthy adults with either evening (ET) or non-evening (MT/IT) chronotypes, in the self-reported scores in the standard tools used for screening for ADHD and deficits in executive functioning, the ASRS-6 and BRIEF-A, respectively. The main hypothesis was that given the association between arousal levels and executive functioning, self-report based screening tools for adult ADS and executive functioning may reflect chronotype-related traits in healthy individuals even in the absence of recognized sleep deficits. We tested the participants’ performance on a visual attention reaction time task and their concurrent self-reported levels of arousal, to quantitate attention maintenance abilities in the two groups during morning hours, time-of-day most relevant to occupational and academic activities. Additionally, day-time sleepiness [Epworth questionnaire (Johns, 1991)] and sleep parameters (actigraphy and sleep diary) were assessed. The outcome measures were compared across groups and tested for correlation with the participants’ scores in the ASRS-6 and BRIEF-A questionnaires.

## Materials and Methods

### Participants

The study was approved by the University of Haifa human experimentation ethics committee. 42 young healthy adults (27 ± 4.3 years old, 29 women) took part in the study. Experiments took place in Haifa, Israel, in the spring–summer months of 2017. The study was advertised in the University of Haifa social media and campus boards. Respondents were first interviewed by an occupational therapist via phone about general health, history of ADHD, learning disabilities and sleep-wake habits. Respondents that affirmed that they were not suggested to have or were never diagnosed as having ADHD/ADD during their childhood or adulthood, had no family history of ADHD and reported no diagnosed sleep disorders, neurological or psychiatric conditions were invited to take part in the study. In total, 44 persons were invited. Two participants, however, dropped out before the commencement of the experiment (starting a new job, illness on the morning of the experiment). Additional exclusion criteria were the use of drugs, heavy alcohol consumption, regular smoking, working in shifts or using medications that might affect arousal or sleep. All the participants were asked not to drink caffeine beverages prior to the experiment session and confirmed they followed this instruction. Participants confirmed they had normal habitual sleep on the pre-experimental night; they were requested to keep a record of bed-time on the pre-experimental and post-experimental nights.

The test session was preceded by ∼30-min accommodation period (journal reading and chatting, no physical activity, no use of devices) in a quiet, air-conditioned (ambient temperature set to 23°C) laboratory room with 150 LUX standard office lighting, to ensure that testing reflects performance at baseline arousal level. During this time the informed consent form was signed. The session started at 8:00 AM (±30 min) and lasted for approximately 35–40 min. It included the VAS assessment for immediate subjective level of arousal, instructions on the simple visual attention task and the tests, followed by filling out the questionnaires. Note, that for ET participants, the testing took place at their non-preferred, off-peak, time of day. All the participants were university students and were paid a sum of 100 NIS (∼26$) to take part in the study.

### Groups

Circadian preferences were prescreened using the Horne–Ostberg MEQ (Horne and Ostberg, 1976) to ensure a range of chronotypes to be represented. Using the standard cut-off score of 41, 21 participants with evening chronotype (26.3 ± 5.1 years old; 16 women; mean MEQ score (31.9 ± 6.27) were included in the ET group and 21 participants with morning/intermediate chronotype [26.7 ± 3.1 years old; 13 women, mean MEQ score (53.57 ± 7.37)] were included in the MT/IT group (distribution of chronotypes in the sample can be seen in Supplementary Figure S1).

### Tests and Questionnaires

During the test session, participants first completed the VAS for the self-assessed current level of alertness, with values ranging from 0 (minimally alert) to 100 (maximally alert). Immediately after the VAS assessment, the participants were engaged in the Deary–Liewald computerized sRT task. The performance in the sRT was used as a measure of sustained visual attention and processing speed (Deary et al., 2011). The program records the response times and the inter-stimulus interval for each trial. A white square was positioned in the center of the screen, set against a blue background. The stimulus to respond to was the appearance of a diagonal cross within the white square. Participants were instructed to respond by pressing a keyboard key, as quickly as possible, each time the cross appeared. The cross remained on the screen until the key was pressed and another cross appeared shortly after. The inter-stimulus interval ranged between 1 and 3 s and was randomly displayed within these boundaries and a total of 80 trials were afforded in two blocks (altogether ∼8–10 min for the sRT session including instructions).

All participants completed the Demographic and General Health questionnaire SF-12 (Ware et al., 1996), BRIEF-A (Roth et al., 2005), ASRS-6 (Adler et al., 2003) and Epworth daytime sleepiness scale (Johns, 1991) pen and pencil questionnaires onsite after the session of reaction time assessment. The ASRS-6 is a brief six questions (rated on a 5-point Likert-type scale) screener that reliably measures frequency of symptoms of inattention and hyperactivity in adults (Kessler et al., 2005; Hesse, 2013). An adjunct reliable screening of adult ADHD is based on the assessment of EFs (Kessler et al., 2010; Ramsay, 2017). The BRIEF-A is composed of 75 items that assess an adult’s EFs or self-regulation in his or her everyday environment on a 3-point scale (Never, Sometimes, or Often) summarized in nine non-overlapping clinical scales. The BRIEF-A subscales are standardized to yield T-scores specific to age and gender norms. Three major index scores are calculated for BRIEF-A: a behavioral regulation index (BRI), a meta-cognition index (MI), and a global executive composite (GEC) score (Roth et al., 2005, 2013). Actigraphy (ActiGraph wGT3X, ActiGraph, LLC) was performed on the post-experimental and not the pre-experimental night to avoid possible bias due to the non-habitual waking times (early morning awakening was imposed by the timing of the neurocognitive tests in the lab, scheduled to 8:00 AM). Actigraphy data was used to verify that participants had no substantial sleep deficits and to assess differences in sleep parameters between the groups. A sleep diary was filled for the nights before and after the day of data collection. The actigraphy data were analyzed using the ActiLife 6 software.

### Data Analysis

Statistical analysis was carried out in SPSS v.25 (IBM Corporation, NY, United States). Independent two-tailed *t*-tests and Mann–Whitney *U* tests for between group comparisons were used to examine the differences in each type of scores and ratings. Analysis of the ASRS-6, Epworth sleepiness questionnaire and BRIEF-A scores used non-parametric statistics. Partial, zero-order Spearman correlations were used to examine the relationships between the variables (Mukaka, 2012). In all analyses, the significance level was set to α = 0.05, two-tailed. *Post hoc* power of group comparisons (*P* = 1 - β err prob), based on the calculated effect sizes and α = 0.05, was estimated using G^∗^Power 3.1 software.

## Results

### Group Comparisons

**FIGURE 1:**
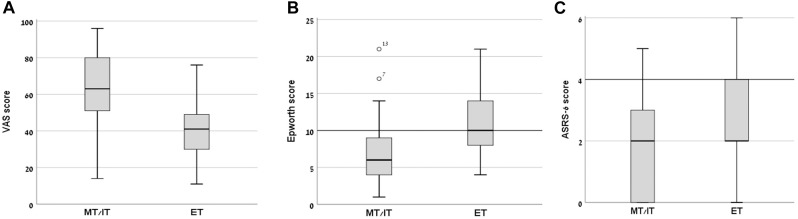
Day-time sleepiness, subjective alertness, and ASRS-6 scores in morning/intermediate type (MT/IT) and evening type (ET) participants. Significant differences between the MT/IT and the ET group were found for: **(A)** VAS scores (ranged between 0–min, 100–max subjective alertness at 8:00 AM), **(B)** Epworth daytime sleepiness scale scores (scores of 10 and above indicate excessive sleepiness), and **(C)** ASRS-6 scores (scores of 4 indicate positive screening for ADHD). Bars, standard deviations of the mean.

Mean subjective arousal levels at 8:00 AM on the day of the experiment, measured using a VAS, differed significantly between the groups (MT/IT group – 63.02 ± 21.40; ET group – 40.76 ± 17.43; *t*(1,40) = 3.69, *p* = 0.001, Cohen’s *d* = 1.14, *P* = 0.56) (Figure 1A). The ET group also had a significantly higher mean rank score in the Epworth sleepiness scale compared to the MT/IT group (MT/IT group 7.19 ± 5.17; ET group 11.48 ± 5.26, Mann–Whitney *U* = 104.5, *p* = 0.003, Cohen’s *d* = 0.822, *P* = 0.718). Moreover, the participants of the ET group demonstrated, on average, mild excessive sleepiness (Figure 1B), with 8/21 participants having scores above 9 [threshold scores 10–15 are considered mild to moderate; scores > 16 indicate severe excessive daytime sleepiness (Johns, 1991)]. Significant differences were found in the reaction times in the Deary–Liewald computerized sRT task [mean RT (msec): MT/IT group 321.47 ± 76.81; ET group 358.94 ± 75.16; Mann–Whitney *U* = 131.0, *p* = 0.039, Cohen’s *d* = 0.493, *P* = 0.33].

In the MT/IT group 1/21, in the ET group 3/21 participants had a score > 4 with all other participants scoring below the clinical threshold (value > 4) on the ASRS-6. However, the ASRS-18 scores showed that even participants with ASRS-6 score above 4 had no clinical-level ADHD symptomatology. In line with the ASRS scores, in the BRIEF-A all participants had a BRI, a MCI, and a GEC score within the age-specific norms (*T* < 65). Altogether, these results indicate that none of the participants was suspected as having ADHD.

The ET group had a significantly higher mean rank score in the ASRS-6 scale compared to the MT/IT group (MT/IT group 1.62 ± 1.59; ET group 2.71 ± 1.62, Mann–Whitney *U* = 139.5, *p* = 0.038, Cohen’s *d* = 0.679, *P* = 0.951) (Figure 1C). No significant differences were found between groups in the BRIEF-A indexes.

Analysis of self-reported bed-time from sleep diary on the pre-assessment night confirmed that bed-time was significantly delayed in the ET group [*t*(1,36) = -2.43, *p* = 0.021, Cohen’s *d* = 0.81, *P* = 0.78], on average 75.33 min later than the MT/IT group (we note that two participants in each group did not keep the sleep diary). Independent samples *t*-tests showed, however, no significant differences between the groups in respect to actigraphy-derived post-experimental night sleep parameters, including sleep efficiency, sleep onset latency, total sleep time, total time in bed, wake after sleep onset and the number of awakenings (Supplementary Table S1). Thus, in the current sample, the differences in performance measures and screening scores of the two chronotype groups may not be ascribed to differences in sleep quality and duration given the participants reports and the limited actigraphy data.

### Correlations

Table 1 summarizes the correlations between the scores on the ASRS-6 and the BRIEF-A major subscales and the measures of chronotype, sleepiness alertness and reaction times, without subdivision to groups. The ASRS-6 scores showed a significant negative correlation with the MEQ score, reflecting that higher ASRS-6 scores were associated with increased eveningness. Higher ASRS-6 scores also showed significant correlations with typical eveningness-related symptoms – higher day-time sleepiness and lower subjective alertness during morning as well as slower reaction times in simple visual RT task. Altogether, these correlations suggest that the ASRS-6 scores reflect not only the level of inattentiveness and hyperactivity, but also the individual’s circadian characteristic of morningness-eveningness the level of subjective alertness and motor vigilance.

**Table 1:** Spearman’s correlations between ASRS-6, BRIEF-A Metacognition index, Behavioral regulation index and Global executive composite subscales, Epworth, MEQ, and VAS scores.

	MEQ	ASRS-6	Epworth	VAS	sRT
MEQ	–	-0.322^*^	0.390^**^	-0.676^***^	-0.373^*^
ASRS-6	–	–	0.384^**^	-0.569^***^	0.320^*^
Epworth	–	–	–	-0.458^**^	0.349^*^
VAS	–	–	–	–	ns
Metacognition index	-0.314^*^	0.693^***^	0.363^**^	-0.548^***^	ns
Initiate	-0.372^**^	0.488^***^	ns	-0.525^***^	0.300^*^
Behavioral regulation index	ns	0.522^***^	0.374^**^	-0.433^**^	ns
Global executive composite	ns	0.647^***^	0.339^**^	-0.496^***^	ns

*MEQ, Morningness-Eveningness Questionnaire; ASRS-6, Adult ADHD Self-Report Scale; VAS, Visual Analog Scale; sRT, simple reaction time. Only significant correlations of r > 0.3 are presented in the table. ^∗^p < 0.05, ^∗∗^p < 0.01, ^∗∗∗^p < 0.001.*

The Metacognition Index (but not the Behavioral Index) of the BRIEF-A also correlated with the individual’s morningness-eveningness. Specifically, the sub-index of “Initiate” function correlated with both the MEQ score and the objectively measured reaction time. Both the Metacognition Index and the Behavioral Index of the BRIEF-A correlated with ASRS-6 score, the Epworth and the VAS arousal scores (Table 1; see Supplementary Table S2 for the same data corrected for multiple comparisons using the Benjamini–Hochberg False Discovery Rate procedure). All nine BRIEF-A subscales had moderate to strong and highly significant correlations with the ASRS-6 score (Supplementary Table S3).

## Discussion

The current exploratory study investigated in young healthy adults the influence of chronotype on neurocognitive performance during morning hours and scores in self-reports about ADS and executive functioning. Significant differences in subjective sleepiness (VAS scale for immediate alertness) and vigilance levels (RT in simple visual attention) at 8:00 AM test sessions were obtained between the ET and MT/IT groups, in line with the literature on the “synchrony” effect (Adan et al., 2012). These differences were in agreement with the significantly increased daytime sleepiness, obtained among the ET group participants (self-report Epworth scale). Scores in the self-report screening tools for symptoms of ADHD, – the BRIEF-A and the ASRS-6, – were below their respective clinical cut-off values. Nevertheless, the ET group scored significantly higher in the mean score of the ASRS-6 scale. Scores of the ASRS-6 and the BRIEF-A-Metacognition Index significantly correlated with chronotype, day-time sleepiness, subjective alertness and motor vigilance in the morning hours. Importantly, these correlations were obtained in young adults with no evident sleep problems. These findings suggest that the standard screening tools for symptoms of ADHD seem to reflect the circadian characteristics, morningness-eveningness, rather than solely the propensity of healthy individuals to show ADHD related symptoms.

Our findings extend the previous notions that chronotype and sleep quality are associated with ADS in people without ADHD diagnosis (Kang et al., 2015; McGowan et al., 2016; McGowan and Coogan, 2018). People with evening chronotypes are susceptible to larger social jetlag (the mismatch between the preferred sleep-wake times and the schedules imposed by academic and social obligations), leading to shorter sleep, – a likely mediator of the ADHD traits in healthy young adults (Gruber et al., 2012; McGowan et al., 2016; McGowan and Coogan, 2018). In the current study, however, no significant differences were found for sleep measures between the ET and MT/IT participants. Thus, evening chronotype by itself may be a significant factor, not necessarily mediated by sleep problems but rather reflecting different diurnal profiles of arousal level. In line with this notion are the findings of studies, suggesting that accounting for circadian profiles can substantially benefit performance and learning in neuro-typical individuals (Bennett et al., 2008; Hahn et al., 2012; Lara et al., 2014) and young adults with ADHD (Korman et al., 2017, 2018) without the need to directly compensate for sleep deficits. Social constrains often impose awakening times close to the body temperature nadir in ETs, when alertness is low (Lack et al., 2009). Recent studies on circadian modulation of brain activation (Gaggioni et al., 2014; Muto et al., 2016) suggest that cortical responses in cognitive tasks are modulated by chronotype.

Healthy college students may be more vulnerable to the negative cognitive effects of later bedtimes and off-peak times of cognitive activity than others (Gao et al., 2019) and there is evidence suggesting that eveningness is a subclinical factor in physical and psychiatric health (Fabbian et al., 2016; McGowan and Coogan, 2018). Individual differences in the magnitude of the social jetlag and associated differences in sleep debt on the days preceding the testing may impact the results of cognitive assessments of adults with ADHD (McGowan et al., 2016), as well as, of healthy adults (McGowan et al., 2016; Barclay and Myachykov, 2017). However, typical ETs have advantages at other times-of-day and during sustained wakefulness over their MT peers (Adan et al., 2012; Barclay and Myachykov, 2017).

Two findings of the current study relate to the question of what constructs are being assessed by the ASRS-6 and the BRIEF-A. The scores of the ASRS-6 and the BRIEF-A (major and minor indexes) were strongly correlated, highlighting a robust association between self-reported ADHD symptomatology and executive functioning in healthy young adults; not only in adults with ADHD (Silverstein et al., 2018). However, it has been suggested that self-reported questionnaires and objective psychometric tests may evaluate different constructs (Emser et al., 2018). In the current study, the reaction times in the sRT task did not correlate with the ASRS-6 or the BRIEF-A indexes, except for the “Initiate” index of the latter. Nevertheless, the MEQ score, i.e., chronotype, correlated with the sRT performance, arguably a measure reflecting executive functioning (Chan et al., 2008).

The results and conclusions drawn from the current study refer specifically to young adults, in their twenties. Chronotype (Roenneberg et al., 2003; Fischer et al., 2017), sleep characteristics (Duffy et al., 2015) and executive abilities (De Luca et al., 2003) change non-linearly during adulthood, thus, links between these parameters are expected to be modified by aging. In agreement with reports on the ubiquity of sleep debt and sleep restriction among college students (Hicks et al., 2001; Lund et al., 2010), participants of both experimental groups in the current study had an indication of sleep debt (total sleep time ∼ 5.8 h, about 1 h less than a physiological norm), however, no differences were found between the means of the total sleep times between the groups and no sleep problems were reported. The single day actigraphy (over the post-test night) is, however, one limitation of the study. It is also likely that a larger acute sleep deficit on the pre-testing night may have occurred in the ET group; the ET group participants went to bed later than their peers by more than an hour but all study participants came to the lab at 8:00 AM (±30 min). Additional limitations of the current study include: (i) the restriction of the sample to university students and the small sample size; (ii) the amalgamation of morning-type and intermediate-type persons under one group of non-evening chronotypes.

Altogether, we report that eveningness, day-time sleepiness and low arousal levels during morning hours can present as subclinical ADHD symptoms in typical young adults with no evident sleep problems. The current findings also raise the possibility that strong eveningness may bias the results of computerized neurocognitive screening for ADHD when administered at morning hours (van der Heijden et al., 2018). In sum, healthy individuals with evening chronotype may experience and demonstrate executive functioning and manifestations of lowered arousal that have been described as typical in ADHD, especially in morning hours; there is a need to carefully consider cognitive symptoms related to evening chronotype that may masquerade as ADHD related symptoms. A differential diagnosis of symptoms of inattention may be helped by cognitive assessments both at peak and off-peak time for the individual.

## Data Availability

All datasets generated for this study are included in the manuscript and/or the Supplementary Files.

## Ethics Statement

This study was carried out in accordance with the recommendations of the University of Haifa human experimentation ethics committee with written informed consent from all subjects. All subjects gave written informed consent in accordance with the Declaration of Helsinki. The protocol was approved by the “name of University of Haifa human experimentation ethics committee.”

## Author Contributions

MK designed the experiments, analyzed the data, and wrote the manuscript. IL, RM-H, and AE-M conducted the screening, collected the data, and preprocessed the data. AK designed the experiments and wrote the manuscript.

## Conflict of Interest Statement

The authors declare that the research was conducted in the absence of any commercial or financial relationships that could be construed as a potential conflict of interest.

## Abbreviations

ADHD: attention deficit and hyperactivity disorder
ADS: attention deficit symptoms
ASRS-6: adult ADHD self-report scale
BRIEF-A: Behavioral Rating Inventory of Executive Functions
ET: evening type
IT: intermediated type
MEQ: morningness-eveningness questionnaire
MT: morning type
sRT: simple Reaction Time
VAS: visual analog scale

## References

[B1] Adan A. Archer S. N. Hidalgo M. P. Di Milia L. Natale V. Randler C. (2012). Circadian typology: a comprehensive review. *Chronobiol. Int.* 29 1153–1175. 10.3109/07420528.2012.719971 23004349

[B2] Adan A. Natale V. Caci H. Prat G. (2010). Relationship between circadian typology and functional and dysfunctional impulsivity. *Chronobiol. Int.* 27 606–619. 10.3109/07420521003663827 20524804

[B3] Adler L. A. Kessler R. C. Spencer T. (2003). *Adult Adhd Self-Report Scale-v1. 1 (ASRS-v1. 1) Symptom Checklist.* New York, NY: World Health Organization.

[B4] Anderson B. Storfer-Isser A. Taylor H. G. Rosen C. L. Redline S. (2009). Associations of executive function with sleepiness and sleep duration in adolescents. *Pediatrics* 123 e701–e707. 10.1542/peds.2008-1182 19336360 PMC4430079

[B5] Bailey S. L. Heitkemper M. M. (2001). Circadian rhythmicity of cortisol and body temperature: morningness-eveningness effects. *Chronobiol. Int.* 18 249–261. 10.1081/cbi-100103189 11379665

[B6] Barclay N. L. Myachykov A. (2017). Sustained wakefulness and visual attention: moderation by chronotype. *Exp. Brain Res.* 235 57–68. 10.1007/s00221-016-4772-8 27624836 PMC5225193

[B7] Bennett C. Petros T. Johnson M. Ferraro F. (2008). Individual differences in the influence of time of day on executive functions. *Am. J. Psychol.* 121 349–361. 18792714

[B8] Brennan A. R. Arnsten A. F. (2008). Neuronal mechanisms underlying attention deficit hyperactivity disorder: the influence of arousal on prefrontal cortical function. *Ann. N. Y. Acad. Sci.* 1129 236–245. 10.1196/annals.1417.007 18591484 PMC2863119

[B9] Bumb J. M. Mier D. Noelte I. Schredl M. Kirsch P. Hennig O. (2016). Associations of pineal volume, chronotype and symptom severity in adults with attention deficit hyperactivity disorder and healthy controls. *Eur. Neuropsychopharmacol.* 26 1119–1126. 10.1016/j.euroneuro.2016.03.016 27150337

[B10] Caci H. Bouchez J. Bayle F. J. (2009). Inattentive symptoms of ADHD are related to evening orientation. *J. Atten. Disord.* 13 36–41. 10.1177/1087054708320439 19387003

[B11] Caci H. Robert P. Boyer P. (2004). Novelty seekers and impulsive subjects are low in morningness. *Eur. Psychiatry* 19 79–84. 10.1016/j.eurpsy.2003.09.007 15051106

[B12] Chan R. C. K. Shum D. Toulopoulou T. Chen E. Y. H. (2008). Assessment of executive functions: review of instruments and identification of critical issues. *Arch. Clin. Neuropsychol.* 23 201–216. 10.1016/j.acn.2007.08.010 18096360

[B13] Coogan A. N. McGowan N. M. (2017). A systematic review of circadian function, chronotype and chronotherapy in attention deficit hyperactivity disorder. *Atten. Defic. Hyperact. Disord.* 9 129–147. 10.1007/s12402-016-0214-5 28064405

[B14] Cunningham J. E. A. Jones S. A. H. Eskes G. A. Rusak B. (2018). Acute sleep restriction has differential effects on components of attention. *Front. Psychiatry* 9:499. 10.3389/fpsyt.2018.00499 30425658 PMC6218409

[B15] De Luca C. R. Wood S. J. Anderson V. Buchanan J.-A. Proffitt T. M. Mahony K. (2003). Normative data from the CANTAB. I: development of executive function over the lifespan. *J. Clin. Exp. Neuropsychol.* 25 242–254. 10.1076/jcen.25.2.242.13639 12754681

[B16] Deary I. J. Liewald D. Nissan J. (2011). A free, easy-to-use, computer-based simple and four-choice reaction time programme: the Deary-Liewald reaction time task. *Behav. Res. Methods* 43 258–268. 10.3758/s13428-010-0024-1 21287123

[B17] Diamond A. (2013). Executive functions. *Annu. Rev. Psychol.* 64 135–168. 10.1146/annurev-psych-113011-143750 23020641 PMC4084861

[B18] Doyle A. E. (2006). Executive functions in attention-deficit/hyperactivity disorder. *J. Clin. Psychiatry* 8 21–26. 16961426

[B19] Duffy J. F. Zitting K. M. Chinoy E. D. (2015). Aging and circadian rhythms. *Sleep Med. Clin.* 10 423–434. 10.1016/j.jsmc.2015.08.002 26568120 PMC4648699

[B20] Emser T. S. Johnston B. A. Steele J. D. Kooij S. Thorell L. Christiansen H. (2018). Assessing Adhd symptoms in children and adults: evaluating the role of objective measures. *Behav. Brain Funct.* 14:11. 10.1186/s12993-018-0143-x 29776429 PMC5960089

[B21] Fabbian F. Zucchi B. De Giorgi A. Tiseo R. Boari B. Salmi R. (2016). Chronotype, gender and general health. *Chronobiol. Int.* 33 863–882. 10.1080/07420528.2016.1176927 27148626

[B22] Fischer D. Lombardi D. A. Marucci-Wellman H. Roenneberg T. (2017). Chronotypes in the Us - Influence of age and sex. *PLoS One* 12:e0178782. 10.1371/journal.pone.0178782 28636610 PMC5479630

[B23] Gaggioni G. Maquet P. Schmidt C. Dijk D. J. Vandewalle G. (2014). Neuroimaging, cognition, light and circadian rhythms. *Front. Syst. Neurosci.* 8:126 10.3389/fnsys.2014.00126 PMC4086398 25071478

[B24] Gao C. Terlizzese T. Scullin M. K. (2019). Short sleep and late bedtimes are detrimental to educational learning and knowledge transfer: an investigation of individual differences in susceptibility. *Chronobiol. Int.* 36 307–318. 10.1080/07420528.2018.1539401 30409040 PMC6377305

[B25] Goodman D. (2009). ADHD in adults: update for clinicians on diagnosis and assessment. *Prim. Psychiatry* 16 38–47. 8912439

[B26] Gruber R. Fontil L. Bergmame L. Wiebe S. T. Amsel R. Frenette S. (2012). Contributions of circadian tendencies and behavioral problems to sleep onset problems of children with ADHD. *BMC Psychiatry* 12:212. 10.1186/1471-244X-12-212 23186226 PMC3534002

[B27] Gruber R. Grizenko N. Schwartz G. Bellingham J. Guzman R. Joober R. (2007). Performance on the continuous performance test in children with ADHD is associated with sleep efficiency. *Sleep* 30 1003–1009. 10.1093/sleep/30.8.1003 17702270 PMC1978386

[B28] Gualtieri C. T. Johnson L. G. (2005). Adhd: is objective diagnosis possible? *Psychiatry* 2 44–53. PMC2993524 21120096

[B29] Hahn C. Cowell J. M. Wiprzycka U. J. Goldstein D. Ralph M. Hasher L. (2012). Circadian rhythms in executive function during the transition to adolescence: the effect of synchrony between chronotype and time of day. *Dev. Sci.* 15 408–416. 10.1111/j.1467-7687.2012.01137.x 22490180 PMC4103784

[B30] Harvey A. G. (2008). Sleep and circadian rhythms in bipolar disorder: seeking synchrony, harmony, and regulation. *Am. J. Psychiatry* 165 820–829. 10.1176/appi.ajp.2008.08010098 18519522

[B31] Hegerl U. Hensch T. (2014). The vigilance regulation model of affective disorders and ADHD. *Neurosci. Biobehav. Rev.* 44 45–57. 10.1016/j.neubiorev.2012.10.008 23092655

[B32] Hesse M. (2013). The ASRS-6 has two latent factors: attention deficit and hyperactivity. *J. Atten. Disord.* 17 203–207. 10.1177/1087054711430330 22262467

[B33] Hicks R. A. Fernandez C. Pellegrini R. J. (2001). Striking changes in the sleep satisfaction of university students over the last two decades. *Percept. Mot. Skills* 93 660–660. 10.2466/pms.2001.93.3.660 11806582

[B34] Horne C. M. Norbury R. (2018). Late chronotype is associated with enhanced amygdala reactivity and reduced fronto-limbic functional connectivity to fearful versus happy facial expressions. *Neuroimage* 171 355–363. 10.1016/j.neuroimage.2018.01.025 29339309

[B35] Horne J. A. Ostberg O. (1976). A self-assessment questionnaire to determine morningness-eveningness in human circadian rhythms. *Int. J. Chronobiol.* 4 97–110. 1027738

[B36] Hsu C. Y. Gau S. S. Shang C. Y. Chiu Y. N. Lee M. B. (2012). Associations between chronotypes, psychopathology, and personality among incoming college students. *Chronobiol. Int.* 29 491–501. 10.3109/07420528.2012.668995 22497432

[B37] Johns M. W. (1991). A new method for measuring daytime sleepiness: the Epworth sleepiness scale. *Sleep* 14 540–545. 10.1093/sleep/14.6.540 1798888

[B38] Kalmbach D. A. Schneider L. D. Cheung J. Bertrand S. J. Kariharan T. Pack A. I. (2017). Genetic basis of chronotype in humans: insights from three landmark GWAS. *Sleep* 40:zsw048. 28364486 10.1093/sleep/zsw048 PMC6084759

[B39] Kang J. I. Park C. I. Sohn S. Y. Kim H. W. Namkoong K. Kim S. J. (2015). Circadian preference and trait impulsivity, sensation-seeking and response inhibition in healthy young adults. *Chronobiol. Int.* 32 235–241. 10.3109/07420528.2014.965313 25286137

[B40] Kessler R. C. Adler L. Ames M. Demler O. Faraone S. Hiripi E. (2005). The World Health Organization Adult ADHD Self-Report Scale (ASRS): a short screening scale for use in the general population. *Psychol. Med.* 35 245–256. 10.1017/s0033291704002892 15841682

[B41] Kessler R. C. Green J. G. Adler L. A. Barkley R. A. Chatterji S. Faraone S. V. (2010). Structure and diagnosis of adult attention-deficit/hyperactivity disorder: analysis of expanded symptom criteria from the Adult ADHD Clinical Diagnostic Scale. *Arch. Gen. Psychiatry* 67 1168–1178. 10.1001/archgenpsychiatry.2010.146 21041618 PMC3131739

[B42] Kooij J. J. Bijlenga D. (2013). The circadian rhythm in adult attention-deficit/hyperactivity disorder: current state of affairs. *Expert Rev. Neurother.* 13 1107–1116. 24117273 10.1586/14737175.2013.836301

[B43] Korman M. Levy I. Karni A. (2017). Procedural memory consolidation in attention-deficit/hyperactivity disorder is promoted by scheduling of practice to evening hours. *Front. Psychiatry* 8:140. 10.3389/fpsyt.2017.00140 28824471 PMC5540945

[B44] Korman M. Palm D. Uzoni A. Faltraco F. Tucha O. Thome J. (2018). ADHD 24/7: circadian clock genes, chronotherapy and sleep/wake cycle insufficiencies in ADHD. *World J. Biol. Psychiatry* 10.1080/15622975.2018.1523565 [Epub ahead of print]. 30234417

[B45] Lack L. Bailey M. Lovato N. Wright H. (2009). Chronotype differences in circadian rhythms of temperature, melatonin, and sleepiness as measured in a modified constant routine protocol. *Nat. Sci. Sleep* 1 1–8. 23616692 10.2147/nss.s6234 PMC3630920

[B46] Lara T. Madrid J. A. Correa Á. (2014). The vigilance decrement in executive function is attenuated when individual chronotypes perform at their optimal time of day. *PLoS One* 9:e88820. 10.1371/journal.pone.0088820 24586404 PMC3929366

[B47] Lund H. G. Reider B. D. Whiting A. B. Prichard J. R. (2010). Sleep patterns and predictors of disturbed sleep in a large population of college students. *J. Adolesc. Health* 46 124–132. 10.1016/j.jadohealth.2009.06.016 20113918

[B48] Manly T. Lewis G. H. Robertson I. H. Watson P. C. Datta A. K. (2002). Coffee in the cornflakes: time-of-day as a modulator of executive response control. *Neuropsychologia* 40 1–6. 10.1016/s0028-3932(01)00086-0 11595257

[B49] Marzinzik F. Wahl M. Kruger D. Gentschow L. Colla M. Klostermann F. (2012). Abnormal distracter processing in adults with attention-deficit-hyperactivity disorder. *PLoS One* 7:e33691. 10.1371/journal.pone.0033691 22457783 PMC3310872

[B50] Matchock R. L. Mordkoff J. T. (2009). Chronotype and time-of-day influences on the alerting, orienting, and executive components of attention. *Exp. Brain Res.* 192 189–198. 10.1007/s00221-008-1567-6 18810396

[B51] May C. P. (1999). Synchrony effects in cognition: the costs and a benefit. *Psychon. Bull. Rev.* 6 142–147. 10.3758/bf03210822 12199309

[B52] McGowan N. M. Coogan A. N. (2018). Sleep and circadian rhythm function and trait impulsivity: an actigraphy study. *Psychiatry Res.* 268 251–256. 10.1016/j.psychres.2018.07.030 30071388

[B53] McGowan N. M. Voinescu B. I. Coogan A. N. (2016). Sleep quality, chronotype and social jetlag differentially associate with symptoms of attention deficit hyperactivity disorder in adults. *Chronobiol. Int.* 33 1433–1443. 10.1080/07420528.2016.1208214 27668457

[B54] Miyake A. Friedman N. P. Emerson M. J. Witzki A. H. Howerter A. Wager T. D. (2000). The unity and diversity of executive functions and their contributions to complex “Frontal Lobe” tasks: a latent variable analysis. *Cogn. Psychol.* 41 49–100. 10.1006/cogp.1999.0734 10945922

[B55] Moreau V. Rouleau N. Morin C. M. (2013). Sleep, attention, and executive functioning in children with attention-deficit/hyperactivity disorder. *Arch. Clin. Neuropsychol.* 28 692–699. 10.1093/arclin/act051 23934136

[B56] Mukaka M. M. (2012). Statistics corner: a guide to appropriate use of correlation coefficient in medical research. *Malawi Med. J.* 24 69–71. 23638278 PMC3576830

[B57] Muto V. Jaspar M. Meyer C. Kusse C. Chellappa S. L. Degueldre C. (2016). Local modulation of human brain responses by circadian rhythmicity and sleep debt. *Science* 353 687–690. 10.1126/science.aad2993 27516598

[B58] Ottoni G. L. Antoniolli E. Lara D. R. (2012). Circadian preference is associated with emotional and affective temperaments. *Chronobiol. Int.* 29 786–793. 10.3109/07420528.2012.679329 22734579

[B59] Owens J. Gruber R. Brown T. Corkum P. Cortese S. O’brien L. (2013). Future research directions in sleep and ADHD: report of a consensus working group. *J. Atten. Disord.* 17 550–564. 10.1177/1087054712457992 22982880

[B60] Park C. I. An S. K. Kim H. W. Koh M. J. Namkoong K. Kang J. I. (2015). Relationships between chronotypes and affective temperaments in healthy young adults. *J. Affect. Disord.* 175 256–259. 10.1016/j.jad.2015.01.004 25658501

[B61] Ramsay J. R. (2017). Assessment and monitoring of treatment response in adult ADHD patients: current perspectives. *Neuropsychiatr. Dis. Treat.* 13 221–232. 10.2147/NDT.S104706 28184164 PMC5291336

[B62] Roenneberg T. Merrow M. (2016). The circadian clock and human health. *Curr. Biol.* 26 R432–R433. 27218855 10.1016/j.cub.2016.04.011

[B63] Roenneberg T. Wirz-Justice A. Merrow M. (2003). Life between clocks: daily temporal patterns of human chronotypes. *J. Biol. Rhythms* 18 80–90. 10.1177/0748730402239679 12568247

[B64] Rossa K. R. Smith S. S. Allan A. C. Sullivan K. A. (2014). The effects of sleep restriction on executive inhibitory control and affect in young adults. *J. Adolesc. Health* 55 287–292. 10.1016/j.jadohealth.2013.12.034 24602612

[B65] Rossbach S. Diederichs T. Nothlings U. Buyken A. E. Alexy U. (2018). Relevance of chronotype for eating patterns in adolescents. *Chronobiol. Int.* 35 336–347. 10.1080/07420528.2017.1406493 29231764

[B66] Roth R. M. Isquith P. K. Gioia G. A. (2005). *Behavior Rating Inventory of Executive Function - Adult Version (BRIEF-A).* Lutz, FL: Psychological Assessment Resources.

[B67] Roth R. M. Lance C. E. Isquith P. K. Fischer A. S. Giancola P. R. (2013). Confirmatory factor analysis of the Behavior Rating Inventory of Executive Function-Adult version in healthy adults and application to attention-deficit/hyperactivity disorder. *Arch. Clin. Neuropsychol.* 28 425–434. 10.1093/arclin/act031 23676185 PMC3711374

[B68] Schmidt C. Collette F. Cajochen C. Peigneux P. (2007). A time to think: circadian rhythms in human cognition. *Cogn. Neuropsychol.* 24 755–789. 10.1080/02643290701754158 18066734

[B69] Sikstrom S. Soderlund G. (2007). Stimulus-dependent dopamine release in attention-deficit/hyperactivity disorder. *Psychol. Rev.* 114 1047–1075. 10.1037/0033-295x.114.4.1047 17907872

[B70] Silverstein M. J. Faraone S. V. Leon T. L. Biederman J. Spencer T. J. Adler L. A. (2018). The relationship between executive function deficits and Dsm-5-defined ADHD symptoms. *J. Atten. Disord.* 10.1177/1087054718804347 [Epub ahead of print]. 30296883

[B71] Strauss M. Ulke C. Paucke M. Huang J. Mauche N. Sander C. (2018). Brain arousal regulation in adults with attention-deficit/hyperactivity disorder (ADHD). *Psychiatry Res.* 261 102–108. 10.1016/j.psychres.2017.12.043 29291475

[B72] Um Y. H. Hong S.-C. Jeong J.-H. (2017). Sleep problems as predictors in attention-deficit hyperactivity disorder: causal mechanisms, consequences and treatment. *Clin. Psychopharmacol. Neurosci.* 15 9–18. 10.9758/cpn.2017.15.1.9 28138105 PMC5290714

[B73] van der Heijden K. B. Stoffelsen R. J. Popma A. Swaab H. (2018). Sleep, chronotype, and sleep hygiene in children with attention-deficit/hyperactivity disorder, autism spectrum disorder, and controls. *Eur. Child Adolesc. Psychiatry* 27 99–111. 10.1007/s00787-017-1025-8 28689312 PMC5799342

[B74] Vitiello M. V. Smallwood R. G. Avery D. H. Pascualy R. A. Martin D. C. Prinz P. N. (1986). Circadian temperature rhythms in young adult and aged men. *Neurobiol. Aging* 7 97–100. 10.1016/0197-4580(86)90146-6 3960269

[B75] Vogel S. W. N. Bijlenga D. Benjamins J. S. Beekman A. T. F. Kooij J. J. S. Van Someren E. J. W. (2017). Attention deficit hyperactivity disorder symptom severity and sleep problems in adult participants of the Netherlands sleep registry. *Sleep Med.* 40 94–102. 10.1016/j.sleep.2017.09.027 29221785

[B76] Wajszilber D. Santiseban J. A. Gruber R. (2018). Sleep disorders in patients with ADHD: impact and management challenges. *Nat. Sci. Sleep* 10 453–480. 10.2147/NSS.S163074 30588139 PMC6299464

[B77] Ware J. Jr. Kosinski M. Keller S. D. (1996). A 12-Item Short-Form Health Survey: construction of scales and preliminary tests of reliability and validity. *Med. Care* 34 220–233. 10.1097/00005650-199603000-00003 8628042

