# Supplementary material for: Subclinical Scores in Self-Report Based Screening Tools for Attention Deficits Correlate With Cognitive Traits in Typical Evening-Type Adults Tested in the Morning
Source: Front Psychol. 2019 Jun 18;10:1397. doi: 10.3389/fpsyg.2019.01397 (PMC6591277; doi:10.3389/fpsyg.2019.01397)
Supplement: Supplementary file 1 [file Table_1.doc]

**Subclinical scores in self-report based screening tools for attention deficits correlate with cognitive traits in typical evening-type adults tested in the morning**

Maria Korman^a^*, Ishay Levy^a,b^, Rinatia Maaravi-Hesseg^a,b^, Adi Eshed^c^ and Avi Karni^a,b^

*^a^The Edmond J. Safra Brain Research Center for the Study of Learning Disabilities, University of Haifa, Israel; ^b^Laboratory for Human Brain and Learning, Sagol Dept. of Neurobiology, University of Haifa, Israel; ^c^Department of Occupational Therapy, Faculty of Social Welfare & Health Sciences, University of Haifa, Israel.*

* Corresponding author

Maria Korman

The Edmond J. Safra Brain Research Center for the Study of Learning Disabilities,

University of Haifa,

Haifa,

Israel

Email: [k](mailto:jason@tau.ac.il)orman.maria@gmail.com

**Supplementary information**

**
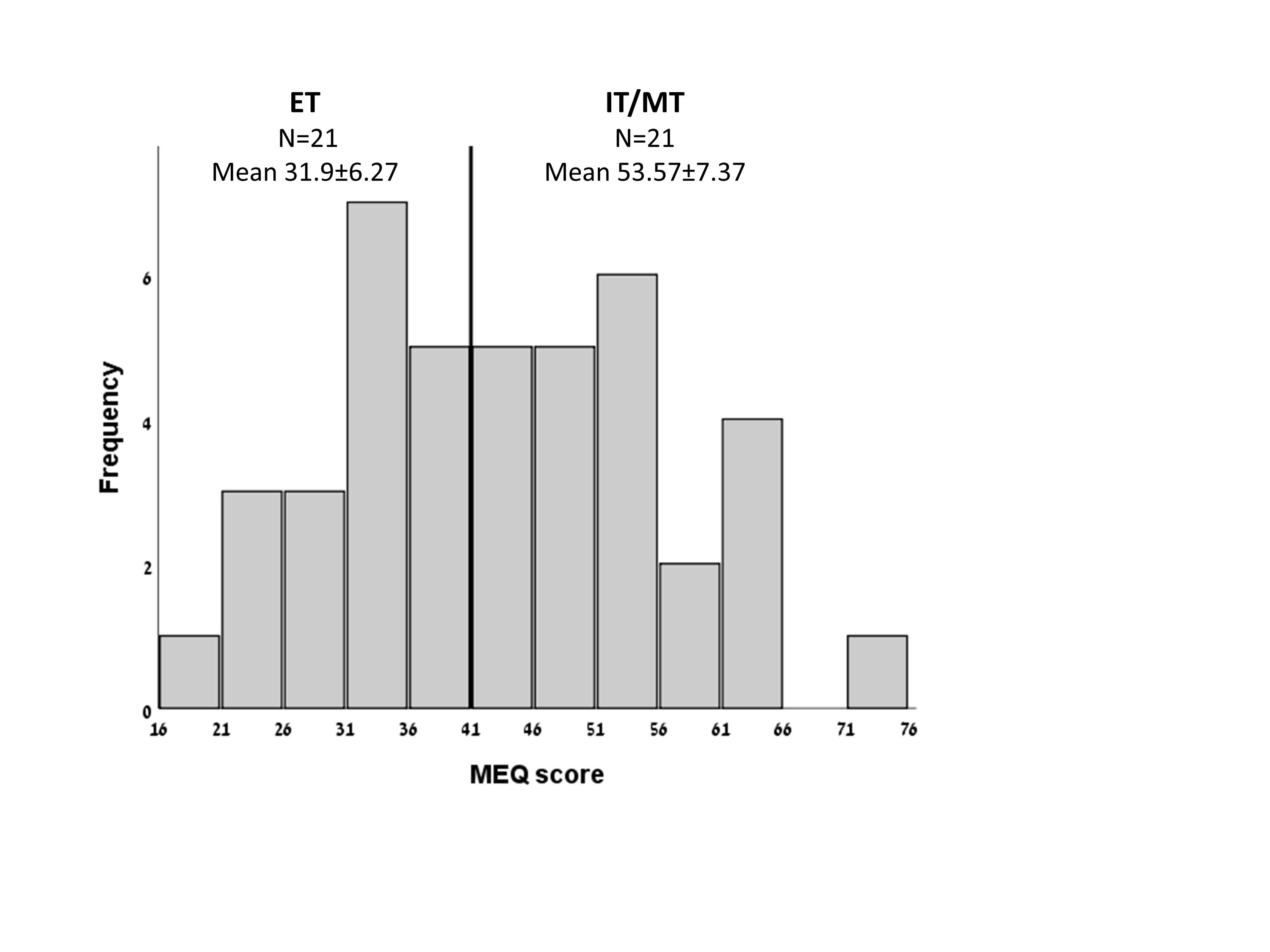
**

**Figure S1.** Distribution of chronotypes in the study sample by continuous MEQ score and the assignment to groups. 42 young adults were invited to take part in the study after answering the morning evening questioner (MEQ); 21 participants were categorized as Morning/Intermediate chronotype (MT/IT group) with an average MEQ score of 53.57 ±7.37 and 21 participants were categorized as evening chronotype (ET group) with an average MEQ score of 31.9±6.27. Vertical line – upper threshold score of eveningness (41).

**Table S1.** Sleep parameters in the MT/IT and the ET groups. Actigraphy, post-test night: Latency, Efficiency, Total time in bed, Total sleep time, Wake after sleep onset, Number of awakenings. Sleep diary: Time in bed on pre-test and post-test nights.

|  | **MT/IT**  **Mean+STD** | **ET**  **Mean+STD** | **t** | **p** |
| --- | --- | --- | --- | --- |
| **Latency** | 8.9±9.5 | 4.7±7.4 | 1.46 | 0.153 |
| **Efficiency** | 82.3±8.5 | 83.8±8.1 | -0.54 | 0.59 |
| **Total Time in Bed** | 429.4±71.7 | 420.4±92.6 | 0.33 | 0.75 |
| **Total Sleep Time** | 353.2±66.5 | 349.5±73.3 | 0.157 | 0.87 |
| **Wake After Sleep Onset** | 72.7±42.7 | 66.2±45.5 | 0.45 | 0.66 |
| **Number of Awakenings** | 20.8±9.5 | 16.6±8.9 | 1.37 | 0.18 |
| **Time-in-bed pre-test night** | 23:47±78 | 01:02±105 | -2.43 | 0.021 |
| **Time-in-bed post-test night** | 23:38±93 | 01:26±87 | -4.21 | 0.001 |

**Table S2.** Spearman’s correlations adjusted using the Benjamini-Hochberg False Discovery Rate (FDR) procedure for multiple comparisons between ASRS-6, BRIEF-A Metacognition index, Behavioral regulation index and Global executive composite subscales, Epworth, MEQ and VAS scores

|  | **MEQ** | **ASRS-6** | **Epworth** | **VAS** | **sRT** |
| --- | --- | --- | --- | --- | --- |
| **MEQ** | - | -0.322* | 0.390** | -0.676*** | -0.373* |
| **ASRS-6** | - | - | 0.384** | -0.569*** | ns |
| **Epworth** | - | - | - | -0.458** | 0.349* |
| **VAS** | - | - | - | - | ns |
| **Metacognition index** | ns | 0.693*** | 0.363** | -0.548*** | ns |
| Initiate | -0.372** | 0.488*** | ns | -0.525*** | ns |
| **Behavioral regulation index** | ns | 0.522*** | 0.374** | -0.433** | ns |
| **Global executive composite** | ns | 0.647*** | 0.339** | -0.496*** | ns |

Abbreviations: MEQ – Morningness-Eveningness Questionnaire, ASRS-6 - Adult ADHD Self-Report Scale, VAS – Visual Analog Scale, sRT – simple reaction time. Only significant correlations of r > 0.3 are presented in the table.

* p < 0.05

** p < 0.01

*** p < 0.001

**Table S3.** Spearman’s correlations of all BRIEF-A subscales with MEQ, ASRS-6, Epworth, VAS and sRT scores

|  | **MEQ** | **ASRS-6** | **Epworth** | **VAS** | **sRT** |
| --- | --- | --- | --- | --- | --- |
| Inhibit | ns | 0.419** | 0.300* | -0.309* | ns |
| Shift | ns | 0.329* | ns | -0.408** | ns |
| Emotional control | ns | 0.355* | 0.306* | -0.321* | ns |
| Self-Monitor | ns | 0.391** | 0.312* | ns | ns |
| **Behavioral regulation index** | ns | 0.522*** | 0.374** | -0.433** | ns |
| Initiate | -0.372** | 0.488*** | ns | -0.525*** | ns |
| WM | ns | 0.649*** | 0.430** | -0.419** | ns |
| Plan/organize | ns | 0.485*** | ns | -0.355** | ns |
| Task Monitor | ns | 0.775*** | ns | -0.575*** | ns |
| Organization | ns | 0.376** | ns | ns | ns |
| **Metacognition index** | -.314* | 0.693*** | 0.363** | -0.548*** | ns |
| **Global executive composite** | ns | 0.647*** | 0.339** | -0.496*** | ns |

MEQ – Morningness-Eveningness Questionnaire, ASRS-6 - Adult ADHD Self-Report Scale, VAS – Visual Analog Scale, sRT – simple reaction time. Only significant correlations of r > 0.3 are presented in the table.

* p < 0.05

** p < 0.01

*** p < 0.001
